# A comparison of early versus late initiation of renal replacement therapy in critically ill patients with acute kidney injury: a systematic review and meta-analysis

**DOI:** 10.1186/cc10061

**Published:** 2011-02-25

**Authors:** Constantine J Karvellas, Maha R Farhat, Imran Sajjad, Simon S Mogensen, Alexander A Leung, Ron Wald, Sean M Bagshaw

**Affiliations:** 1 Division of Critical Care Medicine, University of Alberta, 3C1.12 Walter C. Mackenzie Centre, 8440-122 Street, Edmonton, AB T6G2B7, Canada; 2 Division of Pulmonary and Critical Care Medicine, Harvard Medical School, Brigham and Women's Hospital, 75 Francis Street, PBB - CA 3, Boston, MA 02115, USA; 3 Department of Medicine, Renal Division, Brigham and Women's Hospital, 75 Francis Street, Boston, MA 02115, USA; 4 Department of Anaesthesia, Hvidovre Hospital, DK-2650 Hvidovre, Copenhagen, Denmark; 5 Department of Medicine, Division of General Internal Medicine, University of Calgary, 2500 University Dr. NW, Calgary, AB T2N 1N4, Canada; 6 Division of Nephrology, Department of Medicine, St. Michael's Hospital, University of Toronto, 30 Bond Street, Toronto, ON M5B 1W8, Canada

## Abstract

**Introduction:**

Our aim was to investigate the impact of early versus late initiation of renal replacement therapy (RRT) on clinical outcomes in critically ill patients with acute kidney injury (AKI).

**Methods:**

Systematic review and meta-analysis were used in this study. PUBMED, EMBASE, SCOPUS, Web of Science and Cochrane Central Registry of Controlled Clinical Trials, and other sources were searched in July 2010. Eligible studies selected were cohort and randomised trials that assessed timing of initiation of RRT in critically ill adults with AKI.

**Results:**

We identified 15 unique studies (2 randomised, 4 prospective cohort, 9 retrospective cohort) out of 1,494 citations. The overall methodological quality was low. Early, compared with late therapy, was associated with a significant improvement in 28-day mortality (odds ratio (OR) 0.45; 95% confidence interval (CI), 0.28 to 0.72). There was significant heterogeneity among the 15 pooled studies (*I*^2 ^= 78%). In subgroup analyses, stratifying by patient population (surgical, *n *= 8 vs. mixed, *n *= 7) or study design (prospective, *n *= 10 vs. retrospective, *n *= 5), there was no impact on the overall summary estimate for mortality. Meta-regression controlling for illness severity (Acute Physiology And Chronic Health Evaluation II (APACHE II)), baseline creatinine and urea did not impact the overall summary estimate for mortality. Of studies reporting secondary outcomes, five studies (out of seven) reported greater renal recovery, seven (out of eight) studies showed decreased duration of RRT and five (out of six) studies showed decreased ICU length of stay in the early, compared with late, RRT group. Early RRT did not; however, significantly affect the odds of dialysis dependence beyond hospitalization (OR 0.62 0.34 to 1.13, *I*^2 ^= 69.6%).

**Conclusions:**

Earlier institution of RRT in critically ill patients with AKI may have a beneficial impact on survival. However, this conclusion is based on heterogeneous studies of variable quality and only two randomised trials. In the absence of new evidence from suitably-designed randomised trials, a definitive treatment recommendation cannot be made.

## Introduction

Acute kidney injury (AKI) is a serious complication of critical illness that is associated with substantial morbidity and mortality [1-7]. Extracorporeal renal replacement therapy (RRT) has long been used as supportive treatment of AKI, and has traditionally focused on averting the life-threatening derangements associated with kidney failure (that is, metabolic acidosis, hyperkalemia, uremia, and/or fluid overload) while allowing time for organ recovery. Observations from a large multinational, multicenter survey found the prevalence of severe AKI supported with RRT in critically ill patients was approximately 6% [7].

A critical decision in the support of critically ill patients with AKI is when to initiate RRT. Data have emerged to suggest that earlier RRT initiation may attenuate kidney-specific and non-kidney organ injury from acidemia, uremia, fluid overload, and systemic inflammation [8,9]. This in turn, may potentially translate into improved survival and earlier recovery of kidney function [9]. Unfortunately, in the absence of refractory acidemia, toxic hyperkalemia and intravascular fluid overload contributing to respiratory failure, there is limited evidence to guide clinicians on when to initiate RRT in critically ill patients with AKI. The question of timing of initiation of RRT (that is, "early" versus "late") has seldom been the focus of high-quality or rigorous evaluation [10-23]. As a consequence, initiatives aimed at identifying the "optimal timing of initiation of RRT" in AKI have been given the highest priority for investigation by the Acute Kidney Injury Network (AKIN) [24,25].

Accordingly, we conducted a systematic review and meta-analysis to determine whether "early" versus "late" initiation of RRT in critically ill patients with AKI is associated with a survival benefit or more favourable renal recovery.

## Materials and methods

This study was conducted and reported according to PRISMA guidelines [26] (Additional File 1).

### Search strategy

We performed a comprehensive search of MEDLINE (1985 to July 2010), PubMed, EMBASE (1985 to July 2010), the Cochrane Central Registry of Controlled Trials, Web of Science, and Scopus to identify randomised trials and cohort studies that assessed the timing of initiation of RRT in critically ill patients with AKI. We restricted our search to clinical studies performed in adult populations and published in the English language. We also excluded studies published prior to 1985 largely to reflect important advances in RRT technology and in critical care support not available in older studies.

We extended our search to include clinical trial registries (Controlled trials *meta*Register) and review of abstracts from selected scientific proceedings (Society of Critical Care Medicine, European Society of Intensive Care Medicine and American Society of Nephrology). The bibliographies of all retrieved articles were also hand-searched.

Our search was based on four search themes using the Boolean operator 'OR' (Additional File 2). The first Boolean heading included keyword/MESH headings describing RRT and its different modalities. The second Boolean heading employed terms describing AKI. The third Boolean heading combined the keywords/MESH headings related to critical illness and its different populations. The fourth Boolean search included terms describing timing or initiation of therapy. The searches were combined by using the Boolean term "AND".

### Study selection

Two reviewers (CK and MF/IS/SM) independently performed an initial eligibility screen of all retrieved titles and abstracts (when available). Those studies reporting original data that specifically mentioned the application of RRT in patients with AKI were selected for further review. Full-text review was independently performed by two reviewers (as above) for the following specific eligibility criteria: 1) observational cohort and/or randomised/quasi-randomised clinical trial (RCT) design; 2) adult critically ill population; 3) diagnosis of AKI; 4) description of factors related to timing of initiation of RRT; and 5) description of mortality and/or clinically relevant secondary outcomes (that is, kidney recovery and/or dialysis independence, duration of RRT, and ICU length of stay).

Disagreements between reviewers were resolved by a third reviewer or by discussion and consensus.

### Data extraction

All data were extracted independently with standardised forms with subsequent discussion of any discrepancies. Data were collected on study characteristics and quality, demographics and baseline characteristics (that is, clinical/biochemical parameters at initiation of RRT), and details of RRT modality (that is, continuous venovenous hemofiltration (CVVH), continuous venovenous hemodialysis (CVVHD), continuous venovenous hemodiafiltration (CVVHDF), and intermittent hemodialysis (IHD)). The primary outcome measure was mortality. Secondary outcomes included: kidney recovery and/or dialysis independence, duration of RRT and ICU length of stay.

### Assessment of methodological quality

Randomised studies were appraised using a modified version of the Jadad score [27]. Evaluation of cohort studies was done in a descriptive fashion similar to previous studies [28], incorporating the reported criteria for RRT initiation, assembly of control groups, comparability of intervention/control arms (that is, baseline characteristics, severity of illness, dialysis modality), and a description of dropouts.

### Data analysis and assessment for bias

Data were analysed by STATA version 11 (StataCorp, College Station, TX. USA) and Comprehensive Meta-analysis version 2 (Biostat Inc., Englewood, NJ, USA) [29]. We assessed and quantified statistical heterogeneity for each pooled summary estimate using Cochran's Q statistic and the *I*^*2 *^statistic, respectively [30]. Pooled analysis was performed using the DerSimonian and Laird random effects model and reported as OR with 95% CIs. Meta-regression analysis was performed to assess for possible sources of heterogeneity according to the following pre-defined variables: criteria used to initiate RRT (that is, creatinine, urea, or other), severity of illness (Acute Physiology and Chronic Health (APACHE II) score), type of critical care unit (mixed medical/surgical vs. surgical alone), and study design (observational vs. RCT). Publication bias was assessed using Egger's regression model, and visualised with a funnel plot [31].

## Results

### Trial selection

**Figure 1:**
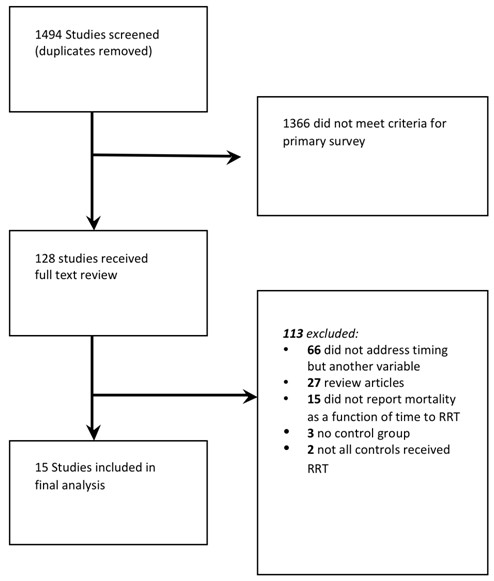
**Outline of study selection process**.

A total of 1,494 citations were identified (Figure 1). After primary and secondary screen, 15 studies fulfilled all criteria for final analysis (13 articles and 2 abstracts).

### Trial characteristics

We found two randomised trials [10,32], four prospective cohort studies [21,33-35], and nine retrospective cohort studies [13,15,36-42]. Of these, 13 were published as articles in peer-reviewed journals and 2 studies were published as abstracts only [33,35]. Eight studies examined only patients with surgical diagnoses (that is, cardiac, abdominal, and trauma) while the remaining seven studies were from mixed medical/surgical ICUs.

### Assessment of trial quality

Of the two included RCTs, one fulfilled all quality indicators [10] (Table 1), whereas the other did not describe the methods of randomisation or perform analysis by intention to treat [32]. Of the 13 cohort studies, none fulfilled all quality indicators (Table 2). Only five had a prospectively assembled control group [21,33-35,41], four had comparable modes of RRT between the early and late initiation groups [15,38,39,41], and only three studies accounted for withdrawals/loss to follow-up [34,35,38].

**Table 1:** Summary of quality indicators and validity assessment of randomised trials fulfilling inclusion criteria

Randomised control trials	**Bouman **[10]	**Sugahara **[32]
Was the study described as randomised?	Yes	Yes
Was the method used to randomise described and appropriate (table of random numbers, computer generated, and so on)?	Yes	No
Was there a description of withdrawals and dropouts?	Yes	Yes
Was there intention to treat analysis?	Yes	No
Were control and intervention group comparable with respect to disease type and demographics?	Yes	No
Were the control and intervention groups comparable with respect to disease severity?	Yes	Yes
Was dialysis type comparable between groups in terms of dose, solution used, filtration vs dialysis, and type of membrane?	Yes	Yes

**Table 2:** Summary of quality indicators of non-randomised studies fulfilling inclusion criteria

Observational Study	**Sabater **[33]	**Bagshaw **[34]	**Gettings **[15]	**Elahi **[38]	**Demirkilic **[13]	**Liu **[21]	**Andrade **[36]	**Wu **[42]	**Manche **[40]	**Iyem **[39]	**Shiao **[41]	**Carl **[37]	**Bagshaw **[35]
**Were criteria for initiation of RRT clearly defined in each group?**	Yes	Yes	Yes	Yes	Yes	Yes	No	Yes	No	Yes	Yes	Yes	Yes
**Was the measurement of criterion (or lab value) for initiation of RRT reliable?**	Yes	Yes	Yes	Yes	Yes	Yes	No	Yes	No	No	Yes	Yes	Yes
**Was control group prospectively assembled? (vs historical, or case-control)**	Yes	Yes	No	No	No	Yes	No	No	No	No	Yes	No	Yes
**Were control and intervention group comparable with respect to disease type and demographics?**	No	No	Yes	Yes	Yes	Yes	Yes	Yes	Yes	Yes	Yes	Yes	Yes
**Were the control and intervention group comparable with respect to disease severity?**	No	No	Yes	Yes	Yes	No	Yes	Yes	No	Yes	Yes	Yes	Yes
**Was dialysis type comparable between groups in terms of dose, solution used, filtration vs dialysis, and type of membrane?**	No	No	Yes	Yes	No	No	No	No	No	Yes	Yes	No	No
**Was there a description of withdrawals and dropouts?**	No	Yes	No	Yes	No	No	No	No	No	No	No	No	Yes

### Type of renal replacement therapy and criteria used for

Continuous renal replacement therapy (CRRT) was used as the principle modality for RRT in eight studies [10,13,15,32,33,38,39,41], while a combination of IHD and CRRT were used in the remaining studies [21,34-37,40,42] (Table 3). Six studies defined timing of initiation of RRT based on cut-offs in serum urea [15,21,34,35,37,42], two studies based on cut-offs in serum creatinine [33,41], one study based on the Risk, Injury, Failure, Loss, End-stage (RIFLE) criteria [3], and four based on urine output [10,32,38,40]. Three other studies used a composite of factors to designate early initiation [13,36,39]. Eight studies reported duration of RRT [10,13,15,33,34,38-40] (range 1 to 20 days). Seven studies described recovery of kidney function (RRT independence) [10,15,32,34,35,39,41].

**Table 3:** Characteristics of studies included in meta-analysis

**Author**:	Year	Study design	Population	Modality	Early (n)	Late (n)	Early criteria	Late criteria
**Bouman **[10]	2002	Randomised	Cardiac surgery/medical	CVVH	35	36	RRT within 12 hours if Urine Output <30 ml/hr	Urea >40 mmol/l or K >6.5 mmol/L
**Sugahara **[32]	2004	Randomised	Cardiac Surgery	CVVH	14	14	Urine Output <20 ml/hr	Urine Output <30 cc/hr
**Liu **[21]	2006	Prospective Cohort	Medical,Surgery	CRRT/IHD	122	121	Urea <27.1 mmol/L	Urea >27.1 mmol/L
**Sabater **[33]	2008	Prospective Cohort	Medical (Septic Shock)	CVVHF	9	23	Rifle Criteria (Risk, Injury)*	Rifle Criteria (Failure)**
**Bagshaw **[34]	2009	Prospective Cohort	Medical, Surgical	CRRT/IHD	618	619	Urea <24.2 mmol/L	Urea >24.2 mmol/L
**Bagshaw **[35]	2010	Prospective Cohort	Medical, Surgical	CRRT/IHD	117	117	Urea <23 mmol/L	Urea >23 mmol/L
**Gettings **[15]	1999	Retrospective Cohort	Trauma	CAVHD and CVVHD	41	59	Urea <21.4 mmol/L	Urea >21.4 mmol/L
**Elahi **[38]	2004	Retrospective Cohort	Cardiac surgery	CVVH	28	36	Urine Output <100 cc in 8 hrs	K >6 mmol/L, Cr >250 mmol/L
**Dermirkilic **[13]	2004	Retrospective Cohort	Cardiac Surgery	CVVHDF	27	34	Cr >400 μmol/L, Potassium >5.5 mmol/L	Oliguria
**Andrade **[36]	2007	Retrospective Cohort	Medical (ARDS/Sepsis)	IHD/SLED	18	15	On admission	At 24 hours
**Wu **[42]	2007	Retrospective Cohort	Surgical ALF	IHD/CVVH	54	26	Urea < 28.6 mmol/L	Urea >28.6 mmol/L
**Manche **[40]	2008	Retrospective Cohort	Cardiac Surgery	IHD	56	15	Hyperkalemia	U/O <0.5 ml/kg/hour
**Iyem **[39]	2009	Retrospective Cohort	Cardia Surgery	CVVH	95	90	RRT on admission	After 48 hours when anuric
**Shiao **[41]	2009	Retrospective Cohort	Surgery/Trauma	CVVH	51	47	Rifle Criteria (Risk)*	Rifle Injury, Failure**
**Carl **[37]	2010	Retrospective Cohort	Medical (sepsis)	CRRT/IHD	85	62	Urea <35.7 mmol/l	Urea >35.7 mmol/L

Abbreviations: Cr = creatinine (μmol/L); K = potassium (mmol/L).

RIFLE Criteria Risk: Increase in serum Creatinine by 1.5 times or urine output <0.5 ml/kg/hour × 6 hours.

RIFLE Criteria Injury: Increase in serum Creatinine by 2 times or urine output <0.5 ml/kg/hour × 12.

RIFLE Criteria Failure: Increase in serum Creatinine by 3 times or urine output <0.3 ml/kg/hour × 24.

### Mortality

**Figure 2:**
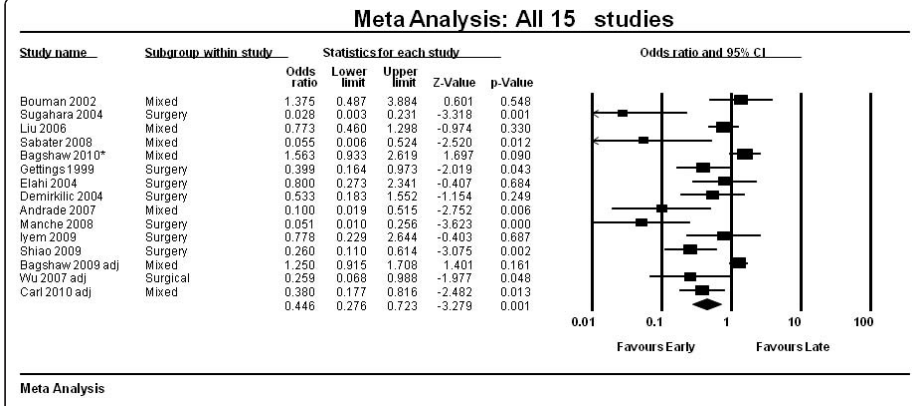
**Forest plot of all 15 studies (Random Effects Model, OR, 95% CI)**.

The OR for 28-day mortality is shown in Figure 2. Overall 28-day mortality across the 15 trials was 53.3% (1,431/2,684). Early RRT initiation was associated with reduced mortality compared to late initiation (pooled OR 0.45; 95% CI, 0.28 to 0.72, *P *< 0.001). However, there was significant statistical heterogeneity (*I*^*2 *^*= 78%, Q 63.7)*.

**Figure 3:**
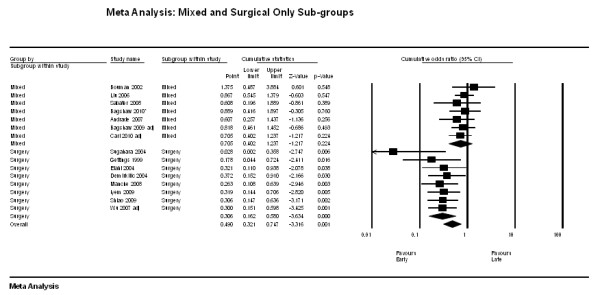
**Forest plot stratified for surgery only (*n *= 8) vs. Medical (mixed, *n *= 7)**.

Subgroup analysis was performed according to type of ICU (mixed vs. surgery only; Figure 3). The overall effect estimate of the surgical group (OR 0.31, 95% CI 0.16 to 0.58, *n *= 8) was not statistically different than that of the mixed group (OR 0.71, 95% CI 0.40 to 1.24, *n *= 7) with a *P*-value of 0.06. There was also no statistical difference in the overall effect estimates between prospective and retrospective studies. There was also no statistically significant effect on the pooled OR for mortality when analysed according to baseline APACHE II scores, creatinine, and urea levels. Therefore, meta-regression analyses with these variables could not account for the large amounts of heterogeneity observed.

### Secondary outcomes

**Figure 4:**
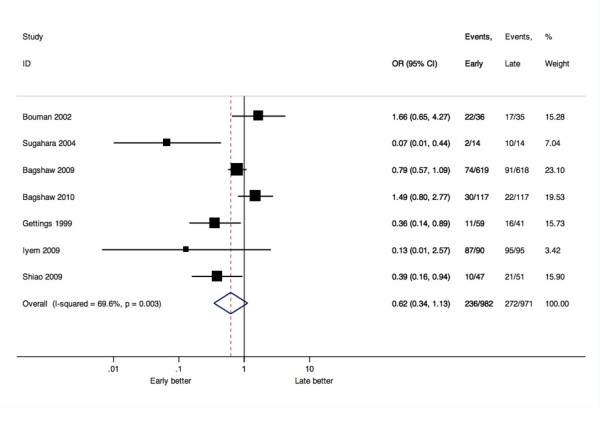
**Forest plot of seven studies reported RRT independence (OR, 95% CI)**.

Five studies [15,32,34,39,41] (of seven reporting data) described a higher rate of kidney recovery to dialysis independence at hospital discharge for patients receiving early RRT (Table 4). Pooled analysis of these seven studies showed a non-significant summary estimate favouring early RRT (OR 0.62, 95% CI 0.34 to 1.13, I^2 ^= 69.6%; Figure 4).

**Table 4:** Baseline characteristics and outcomes in intervention and control groups in 14 studies included in meta-analysis

**Author**:	Year	Creatinine*		Urea*		APACHE II score		Dialysis-Free (%)		Duration of RRT (days)		ICU Length of Stay (days)		Mortality at 28-days (%)	
								
		Early	Late	Early	Late	Early	Late	Early	Late	Early	Late	Early	Late	Early	Late
**Bouman **[10]	2002	5 (4)**	6 (4)**	NR	NR	21.7(5.5)	23.6(8.3)	17	22	5.7	6.6	NR	NR	11/35	9/36
**Sugahara **[32]	2004	256	265	NR	NR	19(2)	18(3)	10	2	NR	NR	NR	NR	2/14	12/14
**Liu **[21]	2006	301	415	16.9	41.0	NR	NR	NR	NR	NR	NR	NR	NR	43/122	50/121
**Sabater **[33]	2008	NR	NR	NR	NR	24(8)	29(9)	NR	NR	6	7	NR	NR	1/9	16/23
**Bagshaw **[34]	2009	230	396	15.0 (5.4)	38.8 (12)	11.1 (3)^§^	10.7 (3)^§^	91	74	4 (2-13)	6 (2-15)	13 (7-24)	13 (6-28)	392/618	380/619
**Bagshaw **[35]	2010	273	489	13.5	38.0	31(9.3)	28.1(6.7)	22	30	NR	NR	12	14	67/117	54/117
**Gettings **[15]	1999	148	238	15.2 (4.6)	33.7 (10)	NR	NR	16	11	17.7	20.2	NR	NR	25/41	47/59
**Elahi **[38]	2004	328	379	23.9 (12)	26.8 (22)	NR	NR	NR	NR	4.61	4.57	8.5	12.5	8/28	12/36
**Dermirkilic **[13]	2004	NR	NR	NR	NR	NR	NR	NR	NR	4.32	4.56	7.8	12.4	8/27	15/34
**Andrade **[36]	2007	583	548	73.9 (6.6)	82.8 (6.9)	24.5 (1.4)	26 (1.2)	NR	NR	NR	NR	20	13.6	3/18	10/15
**Wu **[42]	2007	256	415	16.5 (7)	42.4 (12)	18.2 (5.1)	20.5 (5.3)	NR	NR	NR	NR	NR	NR	34/54	22/26
**Manche **[40]	2008	233	404	14.4 (3.1)	35.2 (18)	NR	.	NR	NR	1.8	6.5	NR	NR	14/56	13/15
**Iyem **[39]	2009	186	256	19.5 (2.7)	24.3 (1.9)	NR	.	95	87	1.6	4.1	2	4	5/95	6/90
**Shiao **[41]	2009	292	336	24.6 (14)	29.2 (14)	18.2 (5.4)	18.8 (6.3)	21	10	NR	NR	NR	NR	22/51	35/47
**Carl **[37]	2010	442	514	23.6 (7.2)	48.9 (10)	24.8 (6.2)	24.7 (6.1)	NR	NR	NR	NR	27	39.1	44/85	42/62

* Continuous variables reported as means and standard deviations when given.

** Bouman *et al*. reported creatinine clearance (ml/minute).

Due to the variability in the reporting of the remaining secondary outcomes of interest and evidence of significant statistical heterogeneity, we did not perform a pooled analysis for RRT duration or ICU length of stay. Rather, we present the data on these secondary outcomes descriptively (Table 4). Seven studies [10,13,15,33,34,39,40] (of eight reported data) described shorter duration of RRT in those receiving early RRT. Five studies [13,35,37-39] (of six reported data) described a reduction in ICU length of stay in those receiving early RRT.

### Publication bias

**Figure 5:**
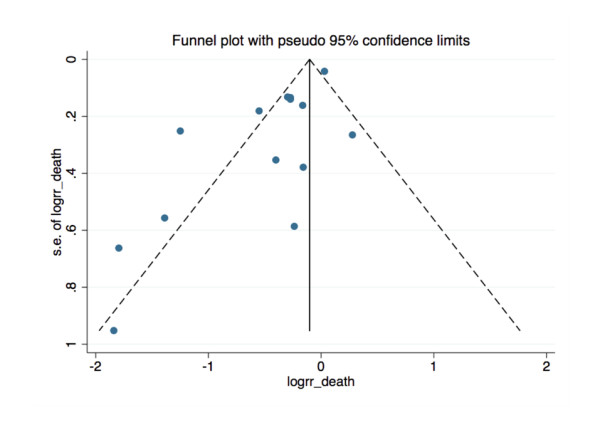
**Funnel plot of all 15 studies**. X-axis is log of risk ratio of death. Y-axis is Standard error of Log Risk ratio of death. Egger's regression (plot not shown): Bias (intercept) -3.19736, *P*-value = 0.00025 (null hypothesis stating no small study effects is REJECTED).

We assessed for publication bias using Egger's linear regression test and found statistical evidence of bias (beta-coefficient of the bias estimate = -3.19, 95% CI = -4.58 to -1.81, *P *= 0.0003). There appears to be publication bias towards smaller studies reporting positive results (that is, mortality benefit associated with early initiation of RRT) (Figure 5).

## Discussion

This systematic review and meta-analysis of 15 unique studies compared "early" versus "late" initiation of RRT in critically ill patients with AKI and suggests that earlier initiation is associated with improved survival. There is insufficient evidence to conclude that kidney recovery to dialysis independence is influenced by the timing of RRT initiation.

To our knowledge, this is the first systematic review to address the question of whether timing of RRT initiation has an important impact on survival and kidney recovery in the critically ill. Previous work on this issue was not specifically focused on critically ill patients supported in an ICU environment [43]. Moreover, in contrast to previous work [43], we intentionally excluded older studies (that is, published before 1985) due to the considerable advances in available technology for providing RRT, the marked demographic transition critically ill populations (that is, older, more comorbid illness, receiving more complex procedures/interventions), and the evolution in general of interventions and technology available to support the critically ill. Accordingly, our systematic review is uniquely focused on how the timing of initiation of RRT impacts survival and kidney recovery in modern ICU practice. Despite these strengths, inferences from our study are limited for two important reasons. First, we found significant statistical heterogeneity. As such, we were unable to calculate effect sizes for all secondary outcomes of interest. We attribute the observed heterogeneity to marked variability between published studies in study design and quality, which we were unable to account for in sensitivity analyses. Second, we found evidence of publication bias towards smaller studies where early initiation of RRT was associated with a survival benefit. As a consequence, the magnitude of the pooled effect estimate may overstate the `true` benefit, if any, of early compared with late RRT initiation.

Our findings are broadly consistent with those reported previously [43]. However, our study more specifically focused on the critically ill and benefited from the recent publication of several additional studies. In a previous meta-analysis [43], Seabra and colleagues explored heterogeneity but found no association between effect estimate and date of publication, RRT modality, sample size, duration of study follow-up, and study quality. Likewise, we could not account for the observed heterogeneity by meta-regression according to patient and population characteristics including type of ICU, severity of illness (baseline APACHE II scores), and metabolic derangements (baseline creatinine and urea levels). Accordingly, the heterogeneity observed is most likely explained by differences in study design (that is, clinical trial vs. cohort study), operational definitions for RRT timing (that is, clinical vs. biochemical criteria) and the inability to account for heterogeneity in clinical practice patterns. Our study has several notable strengths compared to earlier work. First, we have included eight additional clinical studies [32-35,37,39-41]. Second, we excluded studies for which there was no comparable control group [44-46], as well as older studies that have no applicability to current ICU practice [11,16,22]. Third, we have found evidence of publication bias and explain how older reports from smaller studies favouring early RRT may have influenced our summary estimates.

The utilization of RRT in critically ill patients with AKI is relatively common [7,47]. Importantly, the incidence is increasing [48]. These critically ill patients have a risk of death approaching 60% [2,7]. The decision to initiate RRT is a modifiable intervention for these patients; however, it also represents a significant escalation in the complexity and cost of their support. The current uncertainty over the optimal time to initiate RRT is a critical knowledge gap in evidence that has almost certainly contributed to the wide variation in clinical practice. Moreover, this has been further compounded by a lack of consensus and a standardised definition for "early" RRT [24]. There are currently numerous clinical, biochemical, and physiological factors that are considered when deciding to initiate RRT; however, there remains no consensus guidelines or rigorous evidence to guide clinicians on this important issue [24]. This is analogous to the uncertainty regarding the optimal dose-intensity of RRT in critically ill patients with AKI that was largely settled by the recent publication of two large randomised trials [49,50]. A future randomised trial will ideally require broad-based consensus on eligibility criteria and operational definitions for 'early' and 'standard' initiation of RRT in critically ill patients to ensure feasibility and adequate separation of treatment arms. In addition, such a study may benefit from the integration of novel kidney-injury specific biomarkers to aid in the prediction of those who will develop worsening AKI. Understanding methods to further optimise the delivery of acute RRT for critically ill patients with AKI is of utmost importance to improve patient outcomes, guide resource utilization, and rationally deliver standardised care.

## Conclusions

In summary, our systematic review suggests that early institution of RRT in critically ill patients with AKI may have a measurable benefit on survival. However, existing evidence is based on mostly smaller studies with important differences in design and quality, and only two randomised trials. In the absence of novel evidence from a multi-centric suitably-designed randomised trial, conclusive treatment recommendations on the optimal time to initiate RRT remain uncertain. Future investigation must be targeted at defining acceptable "early" RRT criteria and determining whether "early" initiation of RRT, compared with the current standard-of-care, has an important modifying influence on short- and long-term survival and kidney recovery.

## Key messages

• The overall design and quality of studies comparing early versus late initiation of RRT in critically ill patients with AKI is low.

• Earlier initiation of RRT in critically ill patients with AKI may have a beneficial impact on survival.

• A well-designed randomised trial targeting acceptable 'early' compared with "standard" criteria for RRT initiation in homogenous patient populations is needed to definitively determine the effect of RRT timing on patient outcomes.

## Abbreviations

AKI: acute kidney injury; AKIN: Acute Kidney Injury Network; APACHE II: Acute Physiology And Chronic Health Evaluation II; CI: confidence interval; CRRT: continuous renal replacement therapy; CVVH: continuous venovenous hemofiltration; CVVHD: continuous venovenous hemodialysis; CVVHDF: continuous venovenous hemodiafiltration; IHD: intermittent hemodialysis; OR: odds ratio; RCT: randomised control trial; RRT: renal replacement therapy.

## Competing interests

The authors declare that they have no competing interests.

## Authors' contributions

CJK carried out primary study search, extracted data, performed statistical analysis and drafted the manuscript. MF carried out the primary study search, extracted data, performed statistical analysis and tabulated quality indicators of the studies. IS and SM carried out the primary study search and extracted data. AL carried out statistical analysis and helped draft the manuscript. RW helped draft/revise the manuscript. SMB conceived the idea, participated in its design and coordination and drafted/revised the manuscript. All authors read and approved the final manuscript.

## Authors' information

Sean Bagshaw is supported by a Clinical Investigator Award from the Alberta Innovates - Health Solutions (formerly Alberta Heritage Foundation for Medical Research). Alexander Leung is supported by the Alberta Innovates - Health Solutions Clinical Fellowship, the Canadian Institutes for Health Research Fellowship, and the John A. Buchanan Research Chair at the University of Calgary.

## Supplementary Material

Additional File 1 **The PRISMA checklist**. Summary of the completed checklist of quality measures for reporting of systematic reviews and meta-analyses. Click here for file

Additional File 2 **Summary of search strategy**. Detailed summary of search terms and strategy used for systematic literature search. Click here for file
