# Supplementary material for: A comparison of early versus late initiation of renal replacement therapy in critically ill patients with acute kidney injury: a systematic review and meta-analysis
Source: Crit Care. 2011 Feb 25;15(1):R72. doi: 10.1186/cc10061 (PMC3222005; doi:10.1186/cc10061)
Supplement: Additional File 2 — Summary of search strategy. Detailed summary of search terms and strategy used for systematic literature search. [file cc10061-S2.DOC]

**Additional File 2:** Summary of Search Strategy

Two independent reviewers (Reviewer 1: MF, IS, SM and Reviewer 2: CK) searched for both Mesh terms and words appearing in titles or abstracts (tiab).

Our search was based on four search themes using the Boolean operator ‘OR’.

The **first** Boolean heading included the keyword/Mesh headings:

"Renal Replacement Therapy"[Mesh] OR "hemodialysis" OR "hemofiltration" OR "renal replacement"

The **second** Boolean search combined keywords/Mesh headings:

"Kidney Failure, Acute"[Mesh] OR "Oliguria"[Mesh]) OR "Anuria"[Mesh] OR "Acidosis"[Mesh] OR "acute renal failure"[tiab] OR "acute kidney injury"[tiab] OR "oliguria"[tiab] OR "anuria"[tiab] OR "metabolic acidosis"[tiab]

The **third** Boolean search combined keywords/Mesh headings:

"Critical Illness"[Mesh] OR “cardiac surgical procedures”[Mesh] OR "Critical Care"[Mesh] OR "Shock"[Mesh] OR "Sepsis"[Mesh] OR "Multiple Trauma"[Mesh] OR "Rhabdomyolysis"[Mesh] OR "critical illness"[tiab] OR "critically ill"[tiab] OR "critical care"[tiab] OR "shock"[tiab] OR "sepsis"[tiab] OR "polytrauma"[tiab] OR "multiple trauma"[tiab] OR "rhabdomyolysis"[tiab] OR "organ failure"[tiab] or “cardiac surgery” [tiab]
The **fourth** Boolean search combined the keywords/Mesh headings:

"timing"[tiab] or "time"[tiab] OR "initiation"[tiab] OR "start"[tiab] OR "start"[tiab] OR "early"[tiab] OR "late"[tiab]
The Boolean searches were limited to human studies and combined by using the Boolean term “AND”.
